# A Multi-Omics and machine learning platelet-related prognostic signature in multiple myeloma

**DOI:** 10.1007/s00277-026-06867-8

**Published:** 2026-02-28

**Authors:** Xiaojing Li, Qirong Xiao, Kuangfei Wang, Xiaobin Lin, Yu-an He, Jun Peng, Nainong Li, Hai Zhou, Ping Chen

**Affiliations:** 1 Fujian Provincial Key Laboratory on Hematology, Fujian Institute of Hematology, Fujian Medical University Union Hospital, 29 Xinquan Road, Fuzhou, 350001 China; 2 Department of Hematology, Qilu Hospital of Shandong University, 107 Wenhuaxi Road, Jinan, 250012 China; 3 Department of Infectious Disease, Fujian Medical University Union Hospital, Fuzhou, China

**Keywords:** Multiple myeloma, Platelet, Machine learning, Prognosis

## Abstract

While platelets are well-documented contributors to tumorigenesis, their role in multiple myeloma (MM) progression and risk stratification remains underexplored. To assess platelet function in MM and verify the prognostic value of platelet-related genes(PRGs) in patients with multiple myeloma, further providing new ideas for the development of MM. We combined the clinical assessment of platelet activation in MM patients with functional co-culture experiments using MM cell lines (RPMI8226, MM.1 S) to investigate platelet-driven tumor progression. Additionally, an integrated analysis of bulk (GSE124310) and single-cell transcriptomic datasets (GSE6477, TCGA-MM data, GSE4581, GSE24080,and GSE136337) was performed to identify platelet-related prognostic genes (PRGs). Through single-cell RNA sequencing, we identified aberrant erythroid-megakaryocyte components in multiple myeloma and further demonstrated dysfunctional platelet activity that promotes tumor cell proliferation and suppresses apoptosis. Using comprehensive bioinformatic screening across 116 algorithms, we identified a combined forward stepwise Cox and Ridge regression model as optimal and established a 13-gene platelet-related prognostic signature. The genetic risk model effectively stratified MM patients into distinct prognostic groups, with high-risk patients exhibiting poorer outcomes in both training and validation cohorts. Finally, we integrated the genetic risk model and clinically relevant information and visualized it with dynamic Nomogram plots, and the ROC and DCA curves demonstrated that the integrated model had better predictive ability. Our study establishes a significant association between platelet activity and disease progression in MM. The platelet-related prognostic signature we developed is correlated with patient outcomes and may have utility in risk stratification.

**Supplementary Information:**

The online version contains supplementary material available at 10.1007/s00277-026-06867-8.

## Introduction

Multiple myeloma (MM) is a clonal plasma cell malignancy originating from the bone marrow hematopoietic system, characterized by a distinct hypercoagulable state that predisposes patients to thrombotic complications [1–3]. This prothrombotic tendency has been consistently associated with adverse clinical outcomes and poor prognosis in MM patients [4, 5]. Emerging evidence indicates that platelet dysfunction plays a pivotal role in promoting the hypercoagulable state through multiple mechanisms, including pathological platelet hyperactivation, enhanced surface exposure of phosphatidylserine, and disruption of thrombin generation kinetics [6–8]. 

Platelets, which derive from bone marrow megakaryocytes, are increasingly recognized not only for their classical roles in hemostasis and thrombosis, but also for their multifaceted contributions to tumor biology [9, 10]. Recent studies have elucidated the significant involvement of platelets in various aspects of cancer progression, including tumor growth, angiogenesis, and metastasis [11]. In the context of MM, activated platelets may further contribute to disease progression through bidirectional interactions with myeloma cells and the bone marrow microenvironment [12, 13]. Takagi’s research demonstrated that myeloid cell lines (MM.1 S, KMS-11, U266, OPM-2, H929) enhance platelet activation and aggregation. Platelets and their released particles can promote cancer cell proliferation and tumor implantation in vivo by upregulating interleukin-1β (IL-1β) in myeloid cells [14]. These findings indicate that the interaction between tumor cells and platelets plays a crucial role in regulating malignant tumor phenotypes, offering new therapeutic targets for targeting this biological interaction. However, whether platelet-related genes (PRGs) may serve as prognostic markers for multiple myeloma remains unclear.

**Fig. 1:**
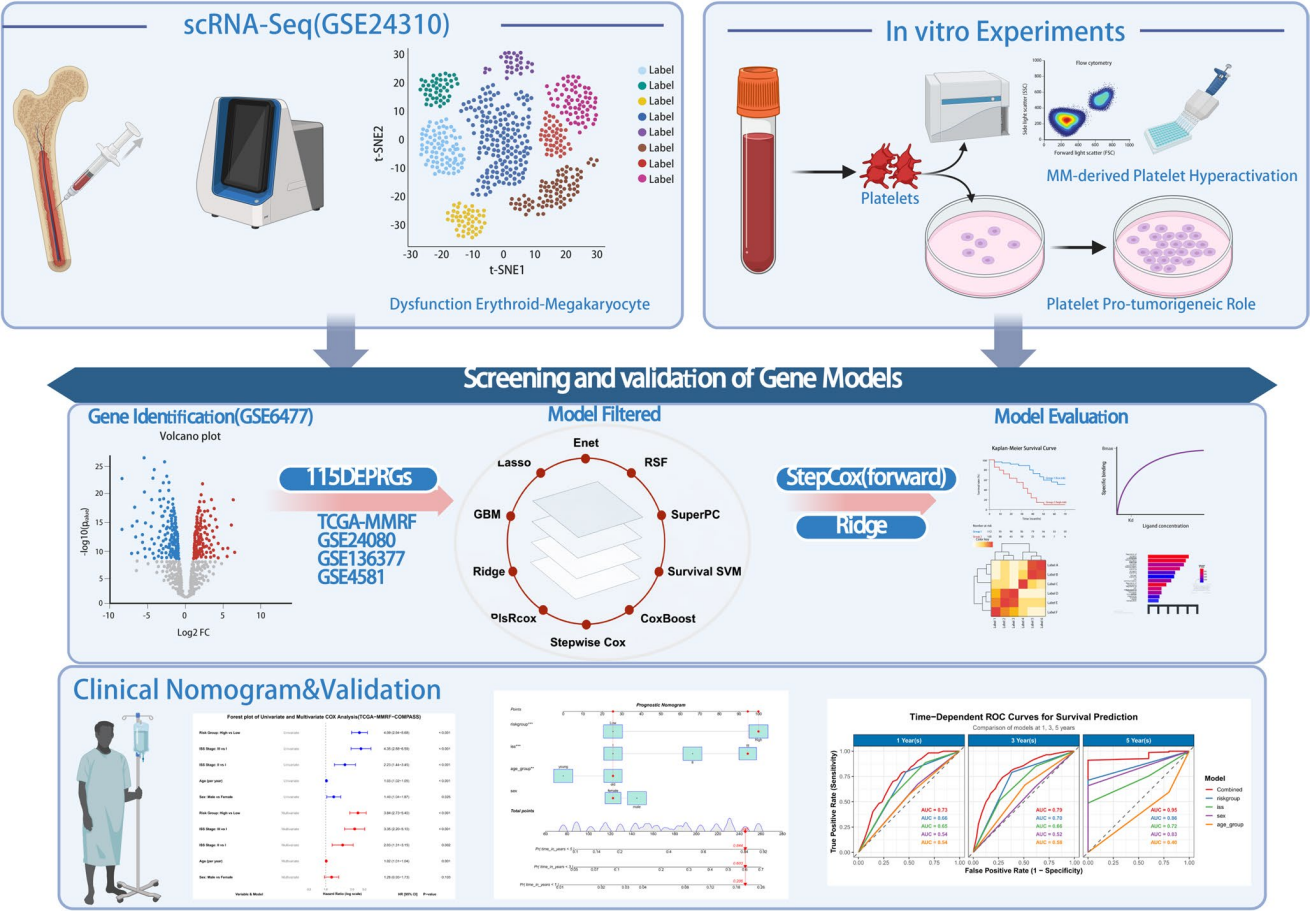
The flow diagram of the study

This study systematically investigates the pathophysiological role of platelet dysfunction in multiple myeloma through an integrated experimental and bioinformatics approach (Fig. 1). We characterize the MM-derived platelet abnormalities, and functional co-culture assays demonstrate their pro-tumorigenic effects on myeloma cell proliferation and apoptosis assays. Furthermore, we establish and validate a platelet-related gene signature through comprehensive bioinformatics analysis of TCGA and GEO datasets to evaluate its prognostic value and clinical utility for MM risk stratification.

## Materials and methods

### Data acquisition and clinical samples

Bulk RNA-seq datasets from multiple myeloma patients with matched clinical data were obtained from the TCGA-MMRF-COMPASS project (https://xenabrowser.net/datapages/), and Gene Expression Omnibus (GEO; accessions GSE4581, GSE136337, GSE24080, GSE6477), while single-cell RNA-seq (scRNA-seq) data of bone marrow mononuclear cells (BMNCs) were acquired from GEO accession GSE124310, including samples from 9 healthy donors (normal bone marrow) and 23 patients with plasma cell disorders (11 smoldering multiple myeloma, 5 monoclonal gammopathy of undetermined significance, and 7 multiple myeloma cases). We enrolled 41 newly diagnosed multiple myeloma patients, 5 multiple myeloma patients who had achieved a complete response (CRMM), and 12 healthy donors from the Department of Hematology at Qilu Hospital of Shandong University, with written informed consent obtained from all participants prior to sample collection.

### Single-Cell RNA-Seq data analysis

We performed initial quality control to remove low-quality cells by filtering based on the number of features (500 < nFeature_RNA < 7000), total counts (nCount_RNA < 30000), and mitochondrial gene percentage (percent.mt < 15). The data were then normalized using the LogNormalize method and analyzed using the Seurat pipeline. To remove doublets, we applied DoubletFinder with parameters pN = 0.25, pK = 0.09, and nExp based on the expected doublet rate of 0.08% per 1000 cells. After PCA (top 2,000 variable genes) and UMAP clustering, cell types were annotated via SingleR (Human Primary Cell Atlas) and classified into hematopoietic lineages (Myeloid, B lineage, Malignant Plasma, etc.). Lineage proportions were quantified using scDC to assess cellular composition dynamics across disease progression. Pseudobulk differential expression analysis of the erythroid-megakaryocyte lineage identified 66 significantly dysregulated genes in MM versus HC (|log2FC| > 0.5, FDR < 0.05), employing a condition-specific model to maximize statistical power given sample size constraints. The biological relevance of these transcriptional alterations was substantiated through functional enrichment analysis (GO and GSEA analysis). Visualizations included UMAP projections, composition bar plots, and standardized heatmaps (ggplot2, version 3.5.2).

### Bulk RNA-Seq data analysis

We curated 480 platelet-related genes (PRGs) from the MSigDB database (Table S1) and identified differentially expressed PRGs (DEPRGs) between healthy donors (*n* = 14) and newly diagnosed multiple myeloma patients (*n* = 101) in the GSE6477 dataset using the “DESeq2” package, with thresholds of |log2FC| > 0.5 and *P* < 0.05. To ensure comparability across cohorts, expression data from TCGA-MMRF-COMPASS, GSE136337, GSE24080 and GSE4581 were integrated and harmonized. Datasets already in transcripts per million (TPM) format were used directly, while raw count data (GSE4581) were log2-transformed. Combined expression matrices were then corrected for batch effects using the ComBat algorithm from the SVA R package.

For prognostic model construction, DEPRGs were evaluated across 116 machine-learning algorithms via the MIME R package. Model performance was assessed using the concordance index (C-index), which identified a combined approach of forward stepwise Cox regression followed by Ridge regression as optimal. The stepwise procedure selected 13 significant genes (KIF22, PCYOX1L, PFN1, MAFF, KIF21B, RAPGEF4, FLNA, HBD, ORM1, DGKE, KIF3B, QSQX1, PHF21A), which were subsequently incorporated into a Ridge regression model to derive the final prognostic signature.

### Platelet preparation and MM cell lines

Platelets were isolated from 1.5 mg/ml EDTA-anticoagulated peripheral blood and bone marrow samples (2 ml) within 30 min of collection. Following gentle inversion, samples were centrifuged at 200 × g for 12 min to obtain platelet-rich plasma. The isolated platelet fraction achieved a purity of > 95%, as determined by flow cytometry analysis. The resulting platelet-rich plasma supernatant was aspirated for downstream processing. We acquired the MM cell lines RPMI8226 from the Cell Bank of the Chinese Academy of Sciences (Shanghai, China) and MM.1 S from the Cell Resource Center, Peking Union Medical College (Beijing, China).

### Assessment of platelet activation status by flow cytometry and Enzyme-linked immunosorbent assay

Markers of platelet activation were assessed based on previously published protocols [15]. Platelets (1 × 10^6^) were stained with PE-conjugated anti-human CD41 (BioLegend) and PE-CY7-conjugated anti-human CD62P (BioLegend) per the manufacturer’s instructions, and after 30 min of incubation at room temperature. All samples were analyzed using a Gallios Flow Cytometer (Beckman Coulter) and Kaluza software. Blood and bone marrow samples were centrifuged (2,000 × g, 10 min) to isolate plasma, which was stored at − 80 °C. P-selectin levels were measured by ELISA (MULTI SCIENCES, Hangzhou, China) following manufacturer’s protocols.

### Platelet - MM cell lines co-culture model

MM cell lines were incubated with platelets at a ratio of 1:100 in complete media (5% CO2, 37 °C). After 96 h, MM cells were collected to analyze platelet-induced proliferation (CCK8 essays, YISHAN, Shanghai, China). After 24 h, the RPMI8226 and MM.1 S cells were obtained by centrifugation at 200 × *g* for 8 min. An Annexin V APC Apoptosis Detection Kit (biogems, American) was used to measure the apoptotic cells according to the instructions.

### RNA extraction and RT-PCR

Total RNA was extracted using a commercial kit (YiShan Biotechnology) and reverse transcribed (Evo M-MLV RT Kit, Accurate Biology) following the manufacturer’s protocols. Gene expression of BCL-2 and BAX was analyzed by qPCR (LightCycler 480, Roche) using GAPDH as reference, with relative quantification by the 2 − ΔΔCt method. The following primer sequences were used:

- BCL-2 : 5′-GGATTGTGGCCTTCTTTGAGTTC-3′(Forward),
- 5′-CTTCAGAGACAGCCAGGAGAAAT-3′(Reverse);
- BAX: 5′-GCTTCAGGGTTTCATCCAGGATC-3′(Forward),
- 5′-ATCCTCTGCAGCTCCATGTTACT-3′(Reverse);
- GAPDH: 5′-GGGAAGCTTGTCATCAATGGAA-3′(Forward),
- 5′-AGAGATGATGACCCTTTTGGCTC-3′(Reverse).

## Statistical analysis

Bioinformatic analysis and visualization in our research were achieved by the R software (version 4.3.0) and its attached packages, and the results of in vitro experiments were presented by Means ± standard deviation (SD) in GraphPad Prism 9.0 software. One-way ANOVA was conducted to analyze the in vitro assays. For each statistical analysis, *p* < 0.05 was regarded as the critical value of a significant difference.

## Result

### Identification of dysfunctional erythroid-megakaryocyte components in multiple myeloma by scRNA-Seq

**Fig. 2:**
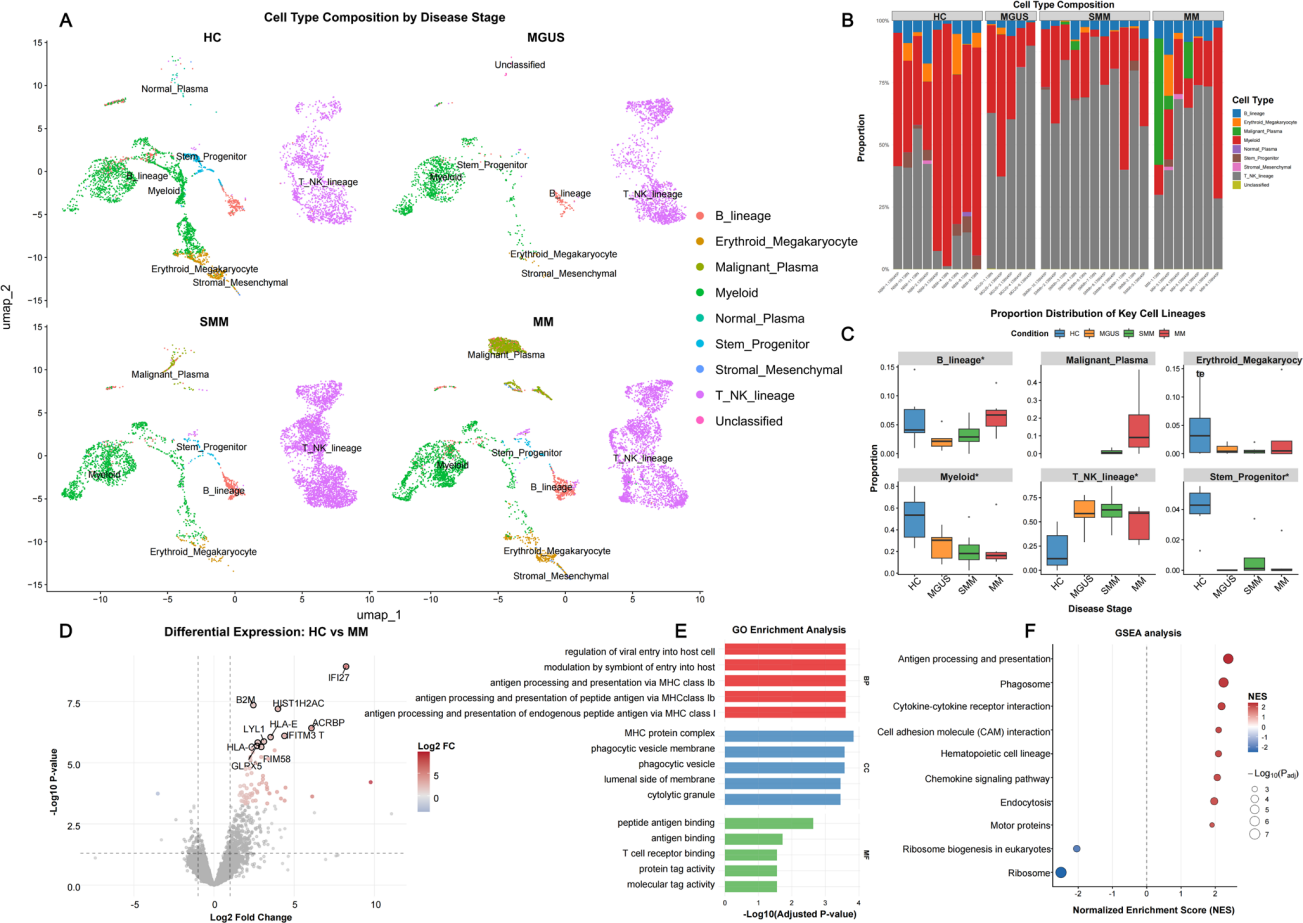
Cell type annotation and visualization for scRNA-seq (GSE124310). (**A**) UMAP projection of bone marrow cells from 9 healthy controls (normal bone marrow) and patients with plasma cell disorders(11 SMM, 5 MGUS, and 7 MM cases), colored by annotated cell type; (**B**-**C**) Cellular composition analysis: per-sample distribution (**B**) and cohort-level summary of key lineages (**C**) across HC, MGUS, SMM, and MM; (**D**–**F**) Downstream analysis of isolated erythroid-megakaryocytic lineage cells: (**D**) Volcano plot of differentially expressed genes (|log₂FC| > 0.5, FDR < 0.05); (E) Gene Ontology enrichment of differentially expressed genes; (**F**) Gene Set Enrichment Analysis (GSEA) results

We performed scRNA-seq analysis on 23,191 high-quality cells from GSE124310 after rigorous quality control and data cleaning. Cell annotation was carried out using a two-step strategy: first, automated fine-grained labeling was conducted with SingleR (v2.8.0) within each disease stage; then, these preliminary annotations were manually aggregated into biologically relevant broad lineages**—**including B_lineage, Malignant Plasma, Myeloid, T_NK_lineage, Erythroid_Megakaryocyte, Stem_Progenitor, and Stromal_Mesenchymal **—**based on marker gene expression and clinical context (Fig. 2A).

To robustly assess cellular composition variations, we utilized scDC for donor-level analysis of cell-type proportions [16]. Fig. 2B–C illustrated shifts in cellular composition across disease progression. Relative to HC, the proportions of B lineage and Erythroid-Megakaryocyte lineages were decreased in MGUS and SMM but increased in MM. The coordinated changes observed in Erythroid-Megakaryocyte and B lineage proportions suggest abnormal dynamics in the erythroid-megakaryocytic compartment during myelomagenesis. Furthermore, due to limited cellularity in MGUS and SMM cohorts, we focused our analysis on the erythroid-megakaryocyte lineage isolated from HC and MM groups, performing pseudobulk-level differential expression analysis between these conditions. As shown in Fig. 2D, the volcano plot displayed differentially expressed genes identified between HC and MM groups. Subsequent functional enrichment analyses (Figs. 2E-F) revealed significant convergence on key biological processes, including immune activation and antigen presentation, as well as cellular communication and adhesion mechanisms.

### Activated platelet promotes myeloma cell proliferation

**Fig. 3:**
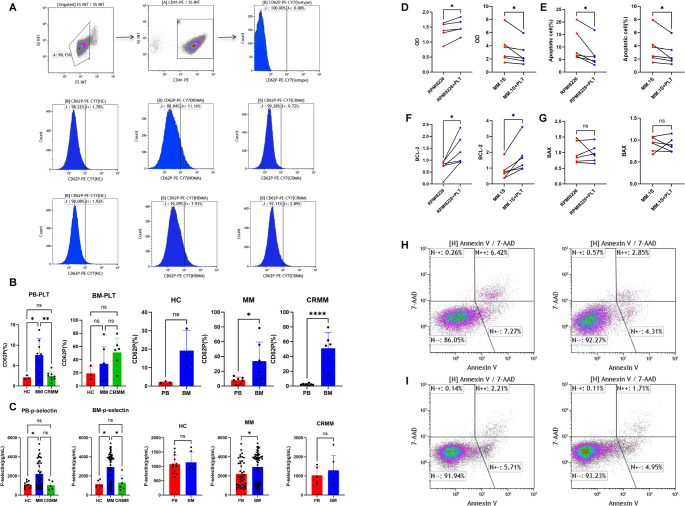
The pro-tumorigenic role of platelets in myeloma through in vitro experiments. Flow Cytometry analysis of platelet activation: (**A**) Schematic representation of the platelet gating strategy; (**B**) Comparative analysis of platelet CD62P expression in peripheral blood and bone marrow from HC and MM patients; (**C**) sP-selectin levels in peripheral blood and bone marrow measured by ELISA in HC and MM groups; (**D**) Effect of MM-derived platelets on proliferation of MM cell lines assessed by CCK-8 assay; (**E**, **H**–**I**) Effect of MM-derived platelets on apoptosis of MM cell lines evaluated by flow cytometry; (**F**–**G**) RT-qPCR analysis of anti-apoptotic BCL-2 (**F**) and pro-apoptotic BAX (**G**) expression levels in RPMI8226 and MM.1 S cell lines. ns, no significant difference, *P < 0.05, **P < 0.01, ****P < 0.0001

The finding that erythroid-megakaryocyte lineage from MM patients exhibited transcriptional alterations, coupled with the established role of platelets in cancer [17], led us to hypothesize functional changes in platelets. This was investigated through detailed analysis of patient-derived platelets. Firstly, we analyzed CD62P expression and soluble P-selectin levels of platelets in samples from HCs and MM patients. Flow cytometry revealed significantly elevated CD62P on peripheral blood platelets in active MM compared to HCs or MM patients in completely remission (CRMM) (*P* < 0.05), while bone marrow platelets showed similar activation across groups. Notably, marrow platelets in MM exhibited higher CD62P than HCs peripheral platelets (*P* < 0.05, Fig. 3A-B). ELISA confirmed increased soluble P-selectin in both plasma and marrow fluid from MM patients (*p* < 0.05, Fig. 3C), with marrow levels exceeding those in plasma during active disease.

To further investigate the tumor-promoting effects of platelets, we established a co-culture system with MM cell lines (RPMI8226 and MM.1 S) and MM-derived platelets. CCK-8 assays indicated that platelet co-culture significantly enhanced MM cell proliferation (*P* < 0.05, Fig. 3D). Flow cytometry revealed a pronounced anti-apoptotic effect, with apoptosis rates decreasing from 10.52% ± 2.55% to 6.78% ± 2.07% in RPMI8226 and from 3.67% ± 0.95% to 2.73% ± 0.70% in MM.1 S (*P* < 0.05, Fig. 3E, H-I). RT-qPCR analysis showed upregulation of the anti-apoptotic gene BCL-2 (*P* < 0.05, Figure F-G), with no significant change in the pro-apoptotic BAX, indicating a platelet-mediated shift in the BCL-2/BAX balance toward survival. These findings demonstrate specific platelet hyperactivation in MM and highlight the role of platelets in promoting tumor cell survival and proliferation within the myeloma microenvironment.

### Development and external validation Platelet-Associated risk signature

In light of the established role of platelets in MM cell proliferation, we evaluated the relevance of PRGs using MM transcriptomic data. From the GSE6477 dataset, differentially expressed PRGs (DEPRGs) were identified (|log2FC| > 0.5, *P* < 0.05). Compared to healthy donors, 72 DEPRGs were upregulated and 43 were downregulated in MM patients (Supplemental Fig. 1 A, C). GO enrichment analysis of these DEPRGs revealed their strong association with platelet activation. Significantly enriched Biological Process (BP) terms included “wound healing,” “hemostasis,” and “coagulation,” while relevant Cellular Component (CC) terms featured “platelet alpha granule lumen,” underscoring the link between the DEPRGs and platelet-related biology.

**Fig. 4:**
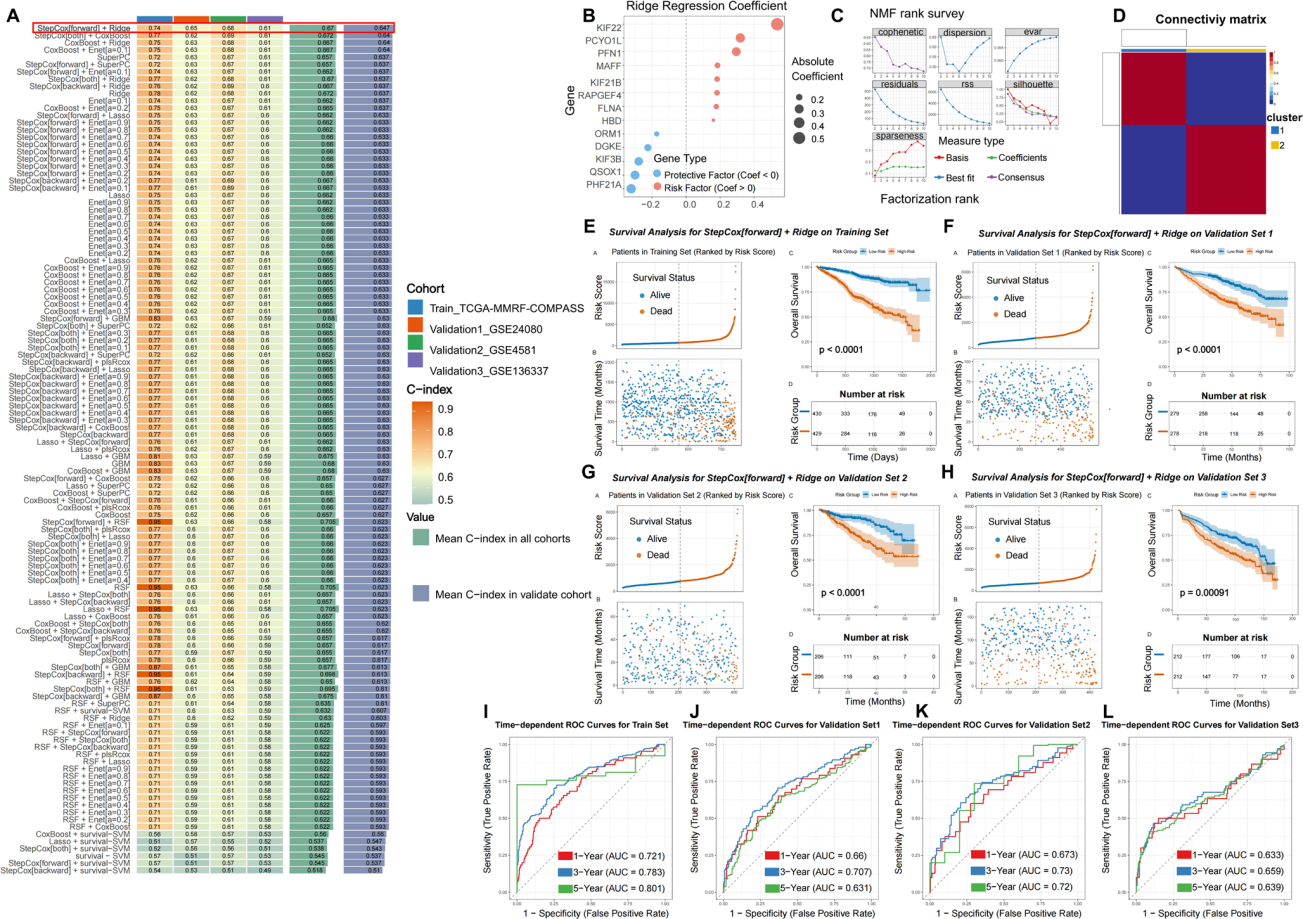
Construction and validation of the platelet-associated risk signature. (**A**) C-indexes of 116 machine-learning algorithm combinations evaluated in the training set (TCGA-MMRP-COMPASS) and three validation sets (GSE24080, GSE4581, GSE136337). (**B**) Coefficients of the 13 final model genes derived from DEPRGs using the StepCox(forward) and Ridge algorithms. (**C**, **D**) NMF consensus clustering of MM samples in the TCGA-MMRP-COMPASS cohort based on the 13-gene signature. (**E**-**L**) Prognostic validation of the risk model. (**E**, **I**) Risk score distribution, Kaplan-Meier survival curves, and time-dependent ROC analysis in the training cohort (TCGA-MMRF-COMPASS). (**F**, **J**), (**G**, **K**), and (**H**, **L**) show corresponding validation in the external datasets GSE24080, GSE4581, and GSE136337, respectively

The 115 DEPRGs expressed by myeloma cells were used to predict MM patients prognosis through 116 combinations of 10 machine learning algorithms in mime (Fig. 4A). The TCGA dataset served as the training set, while the GSE136337, GSE24080 and GSE4581 datasets were used as test sets. Results indicated that the StepCox [forward] + Ridge combination had the highest predictive accuracy, with a C-index of 0.67 across all cohorts and 0.647 in the test cohort. Using StepCox forward selection, 13 key platelet-related genes—KIF22, PCYOX1L, PFN1, MAFF, KIF21B, RAPGEF4, FLNA, HBD, ORM1, DGKE, KIF3B, QSQX1, and PHF21A—were filtered from the 115 DEPRGs (Fig. 4B). Unsupervised clustering based on the expression of these 13 genes identified distinct molecular subtypes of multiple myeloma, yielding two clusters in the training cohort (C1: *n* = 385; C2: *n* = 474, Fig. 4C-D). Subsequently, the 13 DEPRGs were incorporated into a Ridge regression model to generate a prognostic signature. Patients were stratified into high- and low-risk groups according to the median risk score. Kaplan–Meier analysis revealed consistently poorer overall survival in high-risk patients across all four independent cohorts (Fig. 4E-H). The signature demonstrated robust discriminatory performance in predicting 1-year, 3-year, and 5-year overall survival (Fig. 4I–L). In the training set, the AUC values were 0.721, 0.783, and 0.801, respectively. Consistent predictive ability was observed across independent validation cohorts, with AUCs of 0.660, 0.707, and 0.631 in validation set 1 (GSE24080); 0.673, 0.730, and 0.720 in validation set 2 (GSE4581); and 0.633, 0.659, and 0.639 in validation set 3 (GSE136337).

**Fig. 5:**
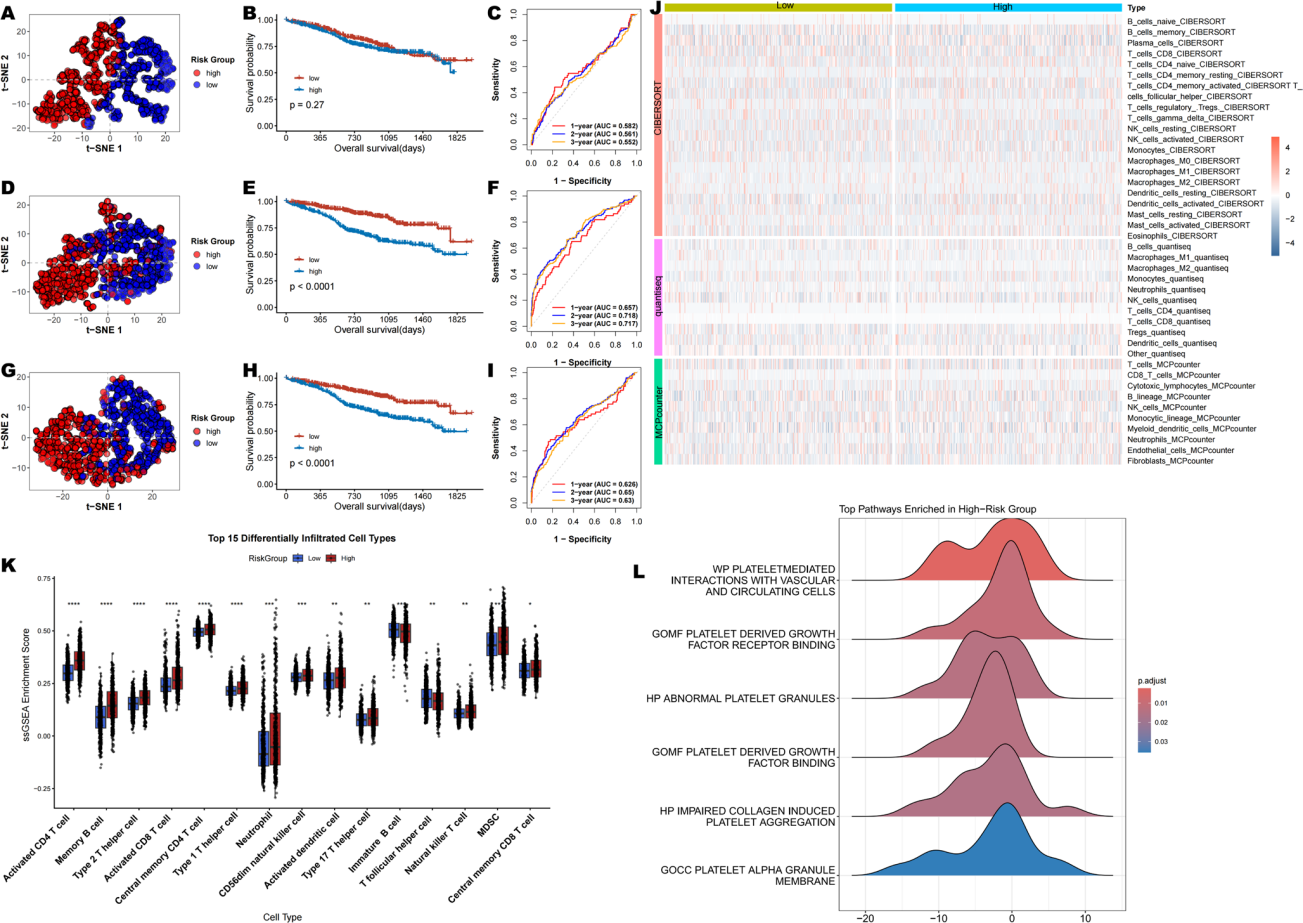
Comprehensive evaluation of prognostic signatures and tumor microenvironment in multiple myeloma. t-SNE visualization, Kaplan–Meier survival analysis, and time-dependent ROC curves for Yu’s signature (**A**–**C**), Huang’s signature (**D**–**F**), and Wang’s signature (**G**–**I**). (**J**) Heatmap of differences in infiltrating immunocytes between distinct risk groups. (**K**) Comparison of scores of immunocytes between two groups based on the ssGSEA algorithm. (**H**) Ridge plot showing the enrichment profiles of platelet-related gene sets in the ranked gene list, as analyzed by GSEA. *P < 0.05, **P < 0.01, ***P < 0.001, ****P < 0.0001

Subsequently, we also made a comparison between our risk signature with other published prediction models in the TCGA-MMRF-COMPASS dataset, including Yu’s signature [18], Huang’s signature [19], Wang’s signature [20]. It was observed that our risk signature had a more noteworthy survival difference compared to other signatures, indicating that our prediction model also had some advantages in horizontal comparison (Fig. 5A-I). Meanwhile, Analysis of the immunological landscape using multiple algorithms revealed a significant difference in immune cell infiltration between the risk groups, with a positive correlation between infiltration levels and risk scores (Fig. 5J). And ssGSEA method-based immune analysis further confirmed that most immunocytes (Type 2 T helper, MDSC cells) were calculated with a higher infiltrating score in patients at high risk (Fig. 5K). Moreover, the ssGSEA enrichment analysis revealed coordinated upregulation of platelet activation, growth factor signaling, and granule function pathways in high-risk patients. This pattern suggests that dysregulated platelet activity converges on pathogenic pathways driving aggressive disease. (Fig. 5L).

### Construction and validation of the combined nomogram

**Fig. 6:**
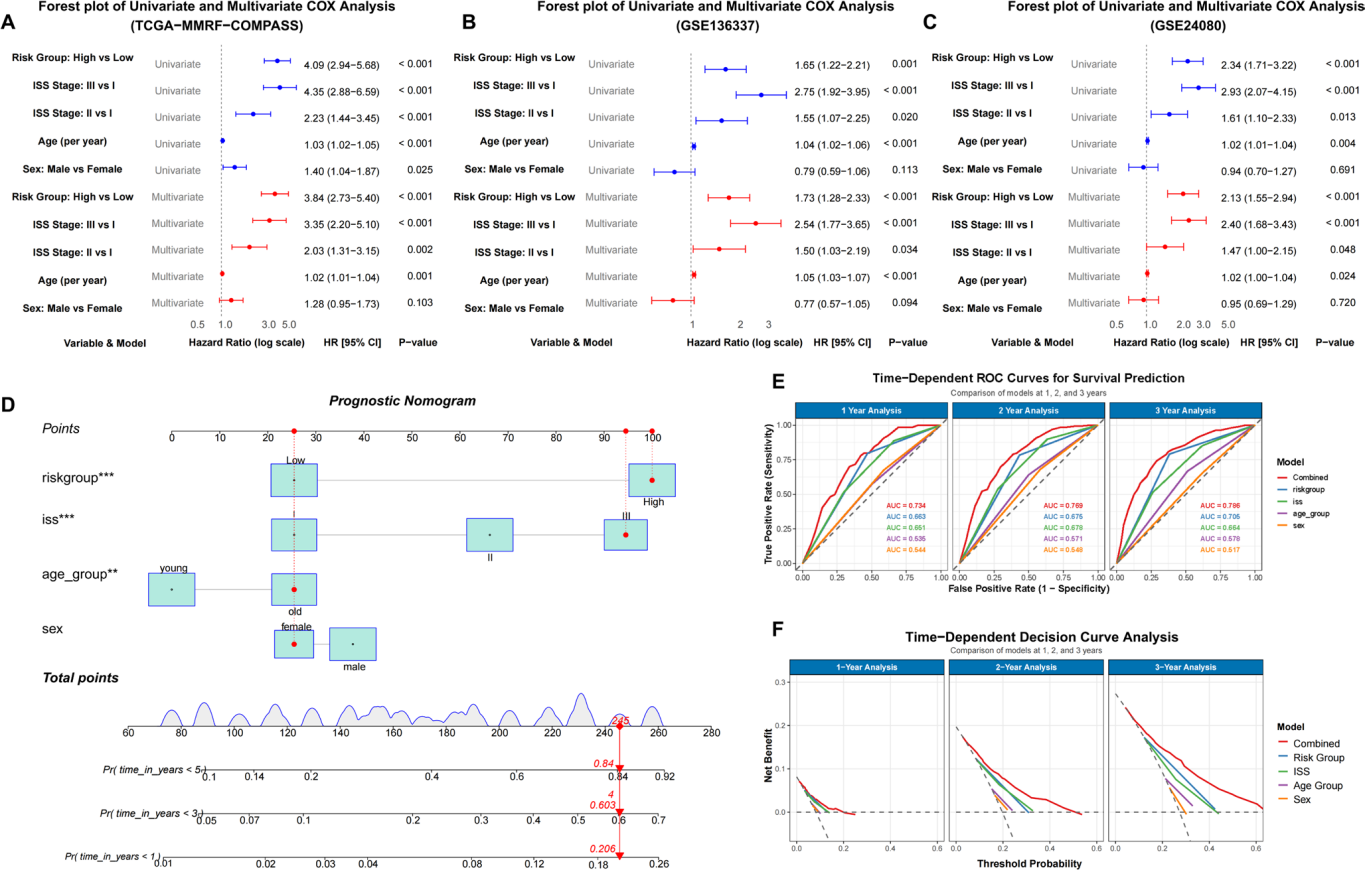
Construction of a Combined Nomogram for Predicting Overall Survival in Multiple Myeloma.**(A-C)** Univariate and multivariate Cox regression analyses of overall survival in the training and validation cohorts. (**D**) A prognostic nomogram integrating sex, age, ISS stage, platelet-related risk score, and total points was developed in the training cohort; age_group young: age < median_age, age_group old: age ≥ median_age (**E**) Time-dependent ROC curves at 1, 2, and 3 years, incorporating various clinical covariates. (**F**) Decision curve analysis (DCA) evaluated the clinical net benefit of the risk score and other prognostic factors

We utilized both univariate and multivariate Cox regression analyses to evaluate the forecasting potential of the platelet-related risk score. Additionally, the influence of various clinical factors such as sex, age, and ISS stage were examined utilizing identical methods in the train and validation datasets (Fig. 6A-C). In the training dataset, the multivariate analysis revealed a HR of 3.84 (95% CI: 2.73–5.40; *p* < 0.001) for the platelet-related risk score. Similarly, in the validation dataset, the multivariate analysis showed a HR of 1.73 (95% CI: (1.28–2.33); *p* < 0.001) in the GSE136337 dataset and 2.13 (95% CI: 1.55–2.94; *p* < 0.001) in the GSE24080 dataset for the platelet-related risk score. Age, sex, ISS stage, and platelet-related risk scores were integrated into combined nomograms to predict 1-, 2-, and 3-year survival rates (Fig. 6D). By incorporating age, sex ISS, and the platelet-related risk score, the nomogram significantly enhanced the accuracy of 1-, 2-, and 3-year survival predictions in the training cohort. The AUC of 1-, 2-, and 3-year improved from 0.651, 0.678, and 0.664 (using ISS alone) to 0.734, 0.769, and 0.786 (using the nomogram), respectively, indicating the superior performance of the nomogram in survival prediction (Fig. 6E). The performance of the nomogram surpassed other metrics when assessed using DCA curves (Fig. 6F).

## Discussion

Multiple myeloma is a hematologic malignancy arising from hematopoietic system abnormalities, often accompanied by abnormal blood cell counts and functions [21]. Previous research has identified peripheral thrombocytopenia as a prognostic risk factor in newly diagnosed MM patients [22, 23]. Given the high blood coagulation tendency and thrombosis susceptibility observed in MM patients [3], we hypothesize that platelet dysfunction may be an associated clinical feature. In this study, we employed single-cell RNA sequencing to identify aberrant erythroid-megakaryocyte components in multiple myeloma. Subsequently, we validated the hyperactivation of platelets in MM patients and elucidated their functional role in promoting tumor cell proliferation and inhibiting apoptosis. Capitalizing on these findings, we derived platelet-related gene sets from the GSEA database to develop a novel platelet-associated risk model, which serves as a robust prognostic tool for MM patients.

Consistent with prior reports, we observed elevated surface expression of CD62P on platelets and increased concentrations of soluble P-selectin in the peripheral blood of patients with multiple myeloma [24, 25]. Interestingly, however, no significant upregulation of CD62P was detected in bone marrow-derived platelets from MM patients when compared to healthy controls or those achieving favorable therapeutic responses. While we implemented standardized protocols to minimize technical variability, platelets remain exquisitely sensitive to collection conditions, anticoagulant choice, and procedural shear stress—factors particularly pronounced during bone marrow aspiration [26–28]. Therefore, we propose that the absence of a detectable difference may reflect not only potential pre-analytical activation of circulating platelets during the procedure but also the heightened susceptibility of platelets to such technical artifacts. Furthermore, inherent variations in marrow sampling may introduce additional noise that obscures underlying biological differences. Moreover, soluble P-selectin levels were significantly higher in the bone marrow supernatant of MM patients than in paired plasma samples (*P* < 0.05), a trend not observed in control groups, suggesting that the MM marrow microenvironment may preferentially promote platelet activation and subsequent P-selectin release [12, 29]. In addition to disease-specific activation, the elevated soluble P-selectin levels in the bone marrow supernatant may also reflect localized concentration effects, such as those resulting from altered plasma viscosity or local protein concentration within the marrow compartment [30, 31]. Therefore, while this finding suggests a distinct microenvironmental profile, further studies are necessary to distinguish true platelet activation from apparent increases due to physicochemical factors.

Beyond their role in shielding circulating tumor cells from hemodynamic shear stress and NK cell-mediated cytotoxicity, platelets exert multifaceted protumor functions through the release of bioactive molecules upon activation [10, 32]. For instance, platelet-derived ADP activates endothelial P2Y₂ receptors, promoting transendothelial migration of tumor cells [33]. Similarly, soluble factors such as TGF-β and lactate dampen effector T-cell function, thereby facilitating immune escape [34, 35]. In the context of MM, platelets and their α-granule contents enhance tumor proliferation and implantation capacity, partly through upregulation of interleukin-1β in myeloma cells [14]. Our in vitro co-culture experiments further corroborate that platelets significantly promote MM cell proliferation and confer resistance to apoptosis. Mechanistically, platelets appear to predominantly engage the BCL-2 anti-apoptotic pathway rather than the BAX pro-apoptotic axis to suppress programmed cell death, although detailed signaling mechanisms remain to be fully elucidated.

Mounting evidence highlights the dynamic interplay between platelets and tumor cells, underscoring the critical role of platelet-related genes not only in facilitating tumor progression but also in shaping disease prognosis [36]. In recent years, platelet transcriptomics has emerged as a highly sensitive and promising methodology for the molecular characterization of malignancies [37, 38]. When integrated with large-scale clinical datasets and machine learning algorithms, this approach enables the precise identification of biomarkers and enhances prognostic prediction [39]. In this study, a prognostic model for multiple myeloma was constructed based on a comprehensive set of 480 platelet-related genes. We developed 116 models using data from multiple MM cohorts and ten commonly used machine learning algorithms. Our results showed that the stepcox (forward) plus Ridge model was the optimal choice through rigorous evaluation. The PRGs is identified as an independent prognostic factor and demonstrates outstanding predictive accuracy across various datasets. Through functional analysis of the 13 platelet-related genes identified by StepCox forward selection, this gene set is found to encompass several key functional aspects of platelet biology. Among them, PFN1 and FLNA are core cytoskeletal proteins that regulate platelet morphology and activation, directly involved in actin dynamics, spreading, and aggregation [40, 41]. DGKE and RAPGEF4 act as important signaling regulators, negatively modulating platelet activation by metabolizing the second messenger DAG and regulating the cAMP pathway, respectively, thereby preventing excessive thrombus formation [42, 43]. Additionally, several kinesin family members such as KIF22 and KIF21B primarily support intracellular granule transport and positioning in anucleate platelets, facilitating secretion and activation [44–46]. At the same time, the set also includes genes such as MAFF, ORM1 and HBD, which are more broadly associated with systemic inflammation, stress responses, or general metabolic processes rather than platelet-specific functions [46–48]. This functional composition suggests that this platelet-related gene signature reflects not only classical platelet activation pathways but also inflammatory and stress signals captured by platelets as circulating “biosensors.” Therefore, its prognostic value may stem from a dual source of information: the functional state of platelets within the tumor microenvironment and the systemic pathophysiological context they reflect, providing a basis for understanding the multifaceted role of platelets in cancer progression.

Using median risk scores, MM patients were effectively stratified into high- and low-risk groups, with the high-risk group exhibiting significantly poorer clinical outcomes. The robust predictive accuracy of the model was supported by ROC analysis and further validated across three independent external cohorts, underscoring its reliability and generalizability. While platelet-related gene signatures have been extensively established as independent prognostic biomarkers in other tumors—including hepatocellular carcinoma [49], breast cancer [50], and pancreatic cancer [51]—their potential remains underexplored in hematologic malignancies. Previous prognostic studies in MM have largely centered on gene expression signatures related to angiogenesis [52], cellular metabolism [19], and apoptosis [53, 54]. To our knowledge, this study represents the first systematic effort to develop a platelet-derived gene expression model for risk stratification in MM. This approach offers a novel and complementary prognostic tool that may enhance clinical assessment and help tailor individualized management strategies for MM patients. Furthermore, single-and multi-factor Cox analyses demonstrated that risk scores serve as a prognostic marker for MM patients. Based on these findings, we developed a dynamic nomogram to predict 1-year, 2-year, and 3-year overall survival probabilities. The validated nomogram exhibited strong predictive capacity in clinical applications.

Finally, our study has several limitations that should be acknowledged. Firstly, the prognostic model was developed and validated primarily using retrospective data from public databases, which may introduce inherent selection biases and unmeasured confounding factors. Therefore, large-scale, multi-center prospective studies are essential to verify the robustness, generalizability, and clinical utility of the proposed platelet-related gene signature. Secondly, while our findings suggest a potential role of platelet-related genes in MM progression, the precise molecular mechanisms remain incompletely elucidated. Our preliminary in vitro experiments were limited in scope, relying primarily on activation markers such as CD62P (P-selectin). Furthermore, despite standardized protocols, the exquisitely sensitive nature of platelets to pre-analytical variables—including collection conditions and procedural shear stress, particularly during bone marrow aspiration—may introduce technical artifacts that complicate mechanistic interpretation. Thus, more comprehensive functional experiments, such as mechanistic co-culture assays, platelet depletion models, and genetic perturbation studies, are warranted to clarify the causal relationships and specific biological pathways involved.

In conclusion, our study establishes a significant association between a platelet-related gene signature and clinical outcomes in multiple myeloma, offering a novel prognostic indicator that may reflect underlying platelet-associated pathophysiology.

## Supplementary Information

Below is the link to the electronic supplementary material.

Supplementary Material 1 (XLSX 15.8 KB)

Supplementary figure 7 Differentially expressed PRGs (DEPRGs) in the GSE6477 cohort and their functional annotation. (PNG 410 KB)

High Resolution Image (TIF 4.36 MB)

## Data Availability

Data can be obtained from the corresponding author upon reasonable request.
