# Supplementary figures and images for: A Multi-Omics and machine learning platelet-related prognostic signature in multiple myeloma
Source: Ann Hematol. 2026 Feb 28;105(4):148. doi: 10.1007/s00277-026-06867-8 (PMC12950032; doi:10.1007/s00277-026-06867-8)

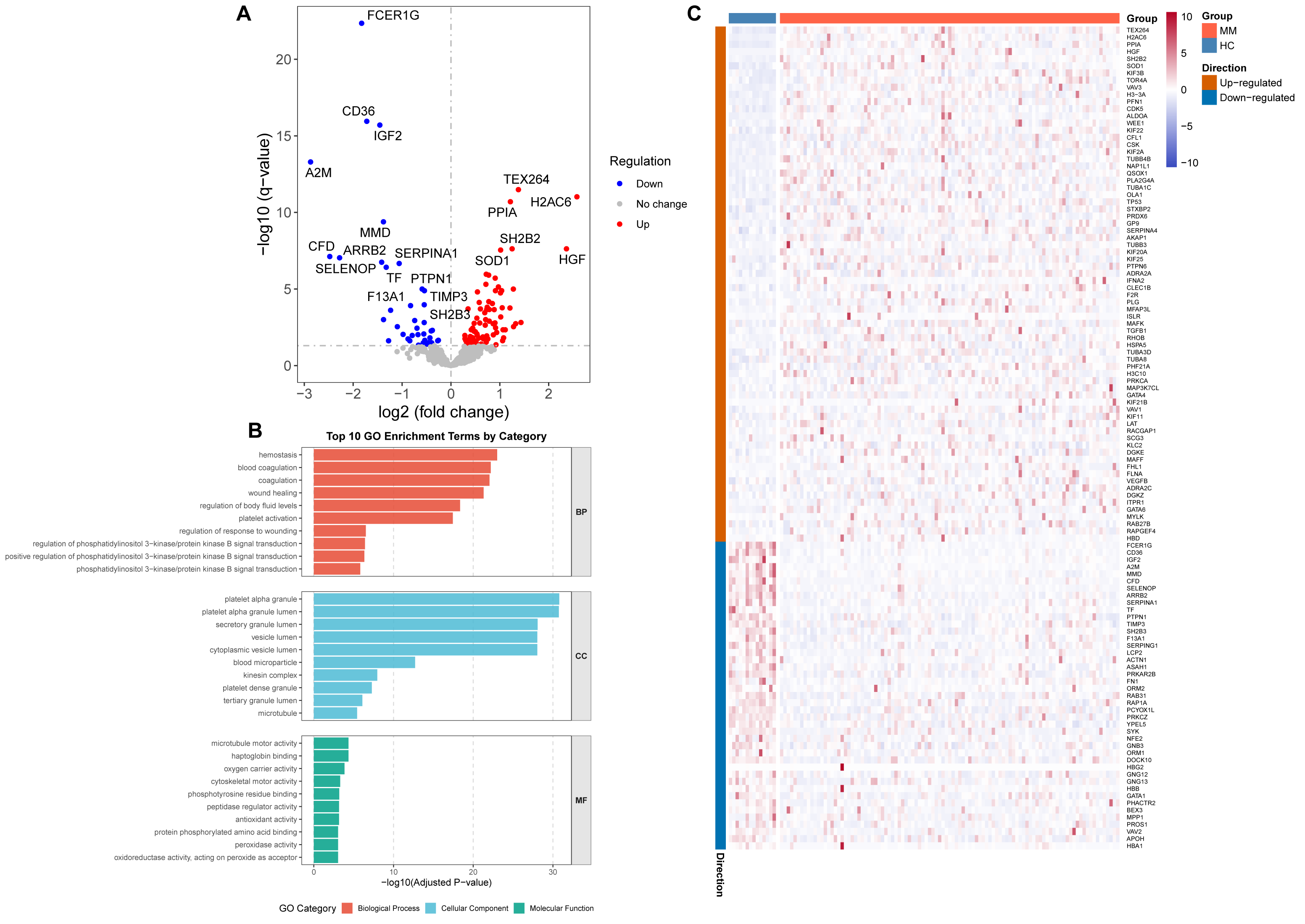

Supplement: Supplementary file 2 — Differentially expressed PRGs (DEPRGs) in the GSE6477 cohort and their functional annotation. (PNG 410 KB) [file 277_2026_6867_Fig7_ESM.png]

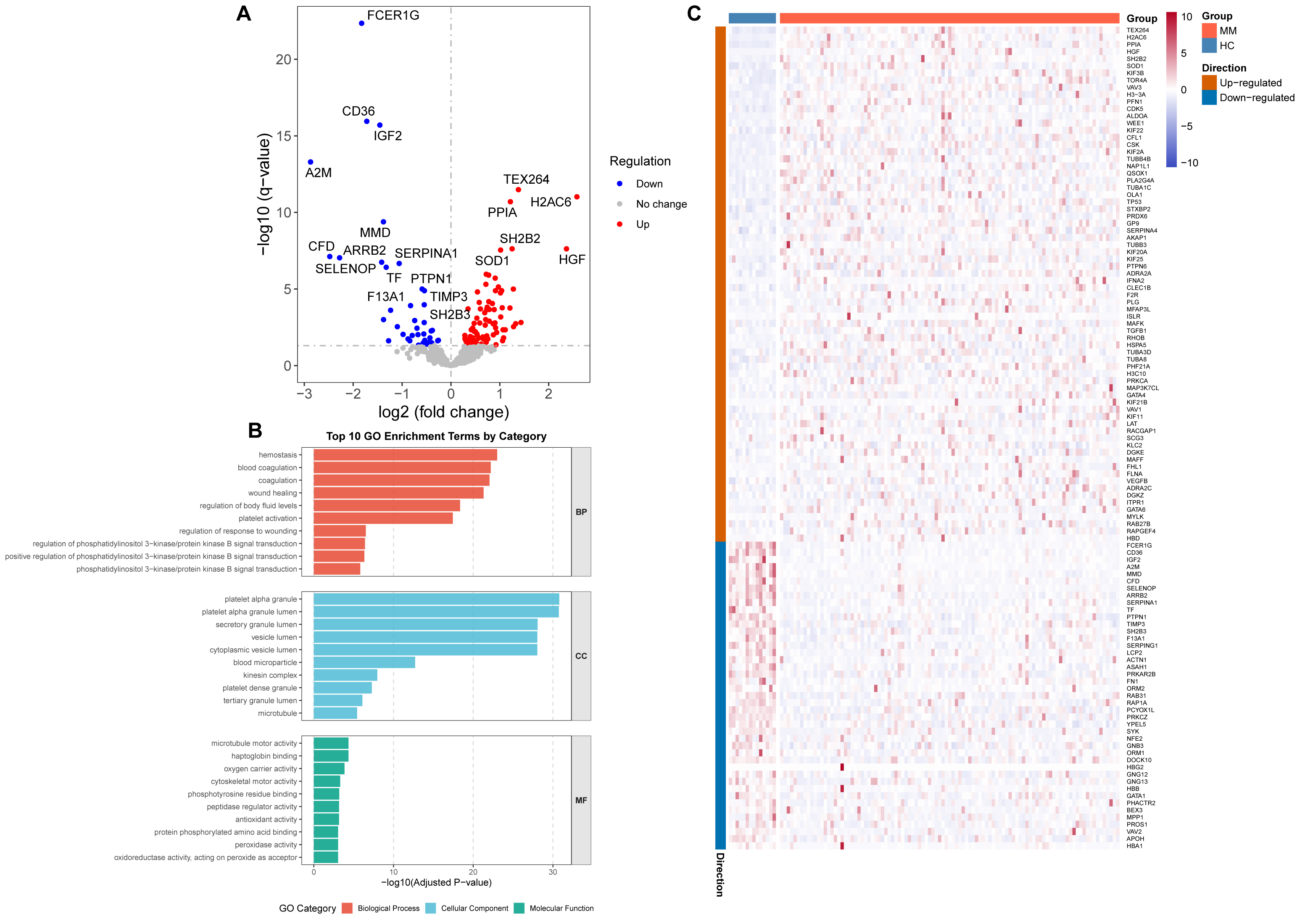

Supplement: Supplementary file 3 — High Resolution Image (TIF 4.36 MB) [file 277_2026_6867_MOESM2_ESM.tif]
